# LRRC8/VRAC volume-regulated anion channels are crucial for hearing

**DOI:** 10.1016/j.jbc.2024.107436

**Published:** 2024-06-04

**Authors:** Deborah A. Knecht, Mariia Zeziulia, Mit B. Bhavsar, Dmytro Puchkov, Hannes Maier, Thomas J. Jentsch

**Affiliations:** 1 Leibniz-Forschungsinstitut für Molekulare Pharmakologie (FMP), Berlin, Germany; 2 Max-Delbrück-Centrum für Molekulare Medizin (MDC), Berlin, Germany; 3 Graduate Program of the Freie Universität Berlin, Berlin, Germany; 4 Department of Otolaryngology, Hannover Medical School, Hannover, Germany; 5 Cluster of Excellence "Hearing4all", Hannover, Germany; 6 NeuroCure Cluster of Excellence, Charité Universitätsmedizin Berlin, Berlin, Germany

**Keywords:** hair cell, anion transport, physiology, osmotic swelling, mouse genetics, VSOR, VSOAC, Swell1, cochlea, volume regulation, VRCC

## Abstract

Hearing crucially depends on cochlear ion homeostasis as evident from deafness elicited by mutations in various genes encoding cation or anion channels and transporters. Ablation of ClC‑K/barttin chloride channels causes deafness by interfering with the positive electrical potential of the endolymph, but roles of other anion channels in the inner ear have not been studied. Here we report the intracochlear distribution of all five LRRC8 subunits of VRAC, a volume-regulated anion channel that transports chloride, metabolites, and drugs such as the ototoxic anti-cancer drug cisplatin, and explore its physiological role by ablating its subunits. Sensory hair cells express all LRRC8 isoforms, whereas only LRRC8A, D and E were found in the potassium-secreting epithelium of the stria vascularis. Cochlear disruption of the essential LRRC8A subunit, or combined ablation of LRRC8D and E, resulted in cochlear degeneration and congenital deafness of *Lrrc8a^-/-^* mice. It was associated with a progressive degeneration of the organ of Corti and its innervating spiral ganglion. Like disruption of ClC-K/barttin, loss of VRAC severely reduced the endocochlear potential. However, the mechanism underlying this reduction seems different. Disruption of VRAC, but not ClC-K/barttin, led to an almost complete loss of Kir4.1 (KCNJ10), a strial K^+^ channel crucial for the generation of the endocochlear potential. The strong downregulation of Kir4.1 might be secondary to a loss of VRAC-mediated transport of metabolites regulating inner ear redox potential such as glutathione. Our study extends the knowledge of the role of cochlear ion transport in hearing and ototoxicity.

The mammalian inner ear is a highly complex structure optimized for detecting sound with exquisite sensitivity in a frequency-dependent manner. Its function crucially depends on ion channels and transporters. These include not only the mechanotransduction channel (1) of sensory hair cells and channels needed for downstream signal transduction, but also transport processes involved in regulating extracellular and intracellular ion concentrations.

The detection of sound by the sensory epithelium of the cochlear organ of Corti (OC) critically depends on extracellular ion homeostasis (2). Mechanotransduction channels, which are located at the tips of cilia of sensory hair cells, are nonselective cation channels. Physiologically, however, they function as K^+^ channels to prevent an influx of Na^+^ and Ca^2+^ into hair cells (2). Otherwise, these ions would have to be extruded from hair cells across their basal membranes against large electrochemical gradients by energy-consuming transporters. For transduction currents to be carried by K^+^, the extracellular solution facing the transduction channel—the endolymph filling the *scala media*—has to have highly unusual properties. It is rich in K^+^ (∼150 mM), but low in Na^+^ (∼1.3 mM) and Ca^2+^ (∼23 μM) and displays a positive voltage (+80 to +120 mV) *versus* normal extracellular space (3). After having entered hair cells by passive diffusion through apical transduction channels, K^+^ exits hair cells again passively through basolateral K^+^-channels prominently including KCNQ4 (Kv7.4) (4). It may then be taken up by adjacent Deiter’s cells through KCC4 K^+^Cl^-^ cotransporters (5). Endolymph production relies on the stria vascularis, a multilayered epithelium in the lateral wall of the cochlea which secretes K^+^ and generates the positive potential of the endolymph (6). Key players in this process include KCNQ1(Kv7.1) potassium channels associated with their β-subunit KCNE1 in the apical, and NKCC1 (SLC12A2) Na^+^K^+^2Cl^-^ cotransporters and ClC-K/Cl^-^ channels associated with their obligatory β-subunit barttin in the basolateral membranes of strial marginal cells, as well as inwardly rectifying Kir4.1 (KCNJ10) K^+^ channels in the underlying intermediate cells. The functional importance of these channels and transporters is evident from mutations which entail deafness in humans or mice (2).

Intracellular ion homeostasis creates appropriate conditions for cellular metabolism and signaling. It is also crucial for cell volume regulation. A key player in the latter process is the volume-regulated anion channel (VRAC) (also known as VSOR, VSOAC, or VRCC) (7, 8, 9). In spite of the importance of cell volume regulation and the recently revealed role of VRAC in the transport of cisplatin (10), a highly ototoxic drug (11, 12, 13), virtually nothing is known about the expression and roles of VRAC in the inner ear. This is probably owed to the fact that the proteins constituting VRAC were unknown until recently (14, 15, 16).

VRACs are heteromers of up to five different leucine rich repeat containing protein 8 (LRRC8) isoforms (14). LRRC8A, the only obligatory subunit (14, 16), associates with any of the other subunits (LRRC8B-E) to form functional channels (14). Both physiological LRRC8A/C heteromers (17) and nonphysiological LRRC8A (18, 19, 20, 21) or LRRC8D (22) homomers are LRRC8 hexamers. The regulation of VRAC channel activity remains poorly understood (8, 23). Opening of LRRC8/VRAC channels is strongly stimulated by cell swelling or low cytoplasmic ionic strength (21, 24, 25, 26). VRACs can also be activated by reactive oxygen species (27, 28) or other, poorly defined pathways. Intriguingly, VRACs not only permeate chloride and other halides, but also small molecules including drugs such as blasticidin (29) and cisplatin (10) and various metabolites including osmolytes like taurine (10, 14, 16), neurotransmitters and amino acids (30, 31), short peptides such as glutathione (32, 33), the immune modulator cGAMP (34, 35) and ATP (36). Physiologically relevant transport of small organic molecules by VRAC/LRRC8 may also occur under resting conditions where VRAC-dependent Cl^-^ currents are difficult to detect (10, 37, 38). The transport of organic substrates depends on the LRRC8 subunit composition, with LRRC8D playing a prominent, but nonexclusive facilitating role (10, 29, 30, 31). Cell volume regulation is only one of many tasks of VRACs, with roles in signal transduction (37) and transepithelial transport (38) being increasingly appreciated.

We now discovered that VRAC is crucial for hearing. Using knock in (KI) mice expressing epitope-tagged versions of LRRC8 subunits (38, 39) we revealed specific expression patterns of all five LRRC8 isoforms in the inner ear. Cochlear hair cells express all LRRC8 isoforms (A-E) and their supporting cells express all isoforms except LRRC8C. Epithelia of the stria vascularis express LRRC8A, D, and E, whereas LRRC8A and C were found in the embedded capillaries. Targeted disruption of the obligatory VRAC subunit LRRC8A in the inner ear led to deafness followed by progressive degeneration of the OC. Whereas single ablation of any of the other LRRC8 isoforms caused no apparent harm, mice disrupted for both LRRC8D and LRRC8E developed similar inner ear pathology as those lacking LRRC8A, albeit with a slower time course. VRACs might directly contribute to ion transport across the stria, but the almost complete loss of Kir4.1, a strial K^+^ channel that is crucial for the generation of the endocochlear potential (EP) (6, 40, 41, 42), points to major indirect effects. Indeed, the hearing loss of VRAC-deficient mice was accompanied by a striking reduction of the EP. Downregulation of Kir4.1, possibly by a lack of a protective substance that normally permeates VRAC, is a major contributor to LRRC8-related deafness.

## Results

### Highly specific expression pattern of VRAC‘s LRRC8 subunits in the inner ear

Both transport properties (10, 30, 31) and regulation (28) of VRAC channels critically depend on the LRRC8 subunit composition. Since our LRRC8 antibodies are not suited for immunohistochemistry (39) we used knock-in mice with various epitopes fused to individual LRRC8 subunits to determine their expression pattern in the inner ear. For LRRC8A and LRRC8B, we also used 5-bromo-4-chloro-3-indolyl-β-D-galactopyranoside (X-gal) staining of mice expressing β-galactosidase under the respective promoters.

**Figure 1:**
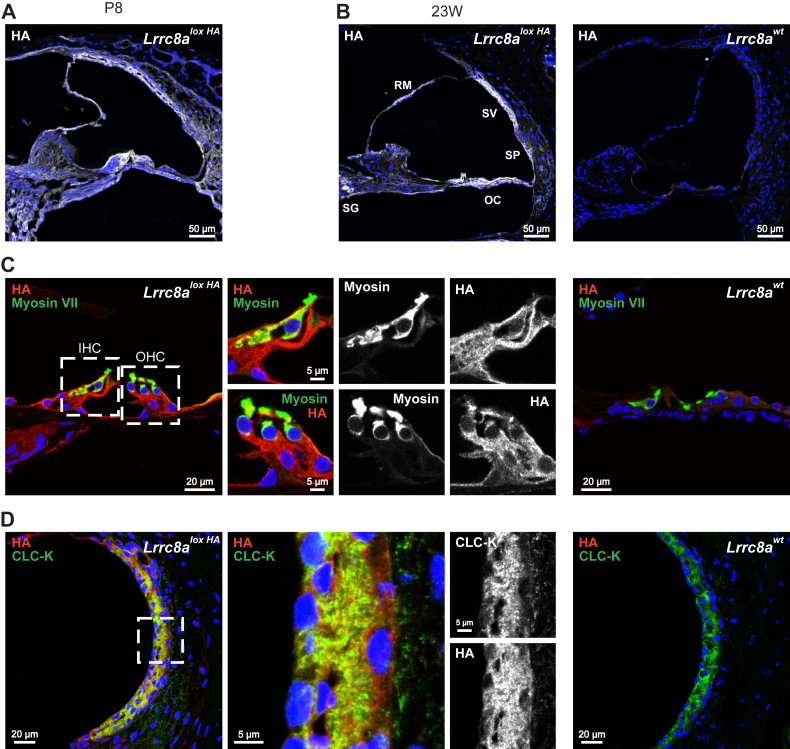
**LRRC8A subunit expression in cochlea.***A*, LRRC8A detected by HA-labeling of *Lrrc8a*^*HA*^^*-lox*^^*/*^^*HA*^^*-lox*^ tissue is widely expressed throughout cochlea of P8 mice. *B*, in adult 23-weeks-old (23W) mice, LRRC8A expression appears more restricted, being found mainly in the organ of Corti (OC), stria vascularis (SV), spiral prominence and outer sulcus cells (SP), Reissner membrane (RM), and spiral ganglion (SG). *C*, in the organ of Corti, HA-tagged LRRC8A is expressed in inner and outer hair cells (IHC and OHC) labeled with hair cell marker myosin VII, as well as in supporting cells. The 23-weeks-old *Lrrc8a*^*HA*^^*-lox*^^*/*^^*HA*^^*-lox*^ mouse. *D*, LRRC8A is expressed in strial cells, labeled with marginal cell marker ClC-K. The 23-weeks-old *Lrrc8a*^*HA*^^*-lox*^^*/*^^*HA*^^*-lox*^ mouse. Images are collected from at least two different mice. WT mice served as negative controls. ClC-K, member of chloride channel family CLC; HA, hemagglutinin.

Cochlear sections from *Lrrc8a^HA-^*^*lox*^*^/HA-^*^*lox*^ mice (38, 43), carrying three hemagglutinin (HA)-epitopes fused to the carboxy terminus of the obligatory subunit **LRRC8A** (sometimes called SWELL1 (16)), as well as X-gal staining of *Lrrc8a*^+/*LacZ*^ sections, revealed wide-spread expression in the inner ear (Figs. 1, *A* and *B* and S1*A*). WT tissue served as a negative control (Fig. 1*B*). There was only a moderate change in expression pattern during postnatal development (Fig. 1, *A* and *B*). In adult mice, immunostaining was most intense in the stria vascularis and in the OC, with prominent labeling of both inner and outer hair cells (IHCs and OHCs) and the underlying supporting cells (Fig. 1*C*). Labeling of the stria diffusely covered the extensively interdigitated marginal and intermediate cell layers (Figs. 1*D* and S1*B*). Whereas this labeling did not allow us to unambiguously attribute LRRC8A expression to one of these cell types, it excluded expression in apical membranes of marginal cells as observed with KCNQ1 K^+^-channels (Fig. S1*B*). The labeling is compatible with LRRC8A being present in basolateral membranes of marginal cells, where they would colocalize with ClC-K/barttin Cl^-^ channels (44), and additionally in intermediate and basal cells as indicated by HA staining extending beyond ClC-K (Fig. 1*D*). Pronounced LRRC8A expression, in particular when examined by X-gal staining (Fig. S1*A*), was also found in the spiral ganglion. Weaker labeling was detected in tympanic border cells, the spiral ligament, Reissner’s membrane, and several types of fibrocytes (Fig. 1*B* and Table 1). In the vestibular organ, LRRC8A was widely expressed in sensory hair cells of both cristae and maculae, endolymph-secreting dark cells, and fibrocytes (Fig. S2, *A*, *B*, and *D*). Similar expression patterns were observed by X-gal staining of *Lrrc8a*^+/*LacZ*^ cochleae (39) (Fig. S2*C*).

**Table 1:** Expression pattern of VRAC subunits in the cochlea

Cell type	LRRC8A	LRRC8B	LRRC8C	LRRC8D	LRRC8E
Inner hair cells	++	++	++	+	++
Outer hair cells	++	++	+	+	++
Supporting cells	++	++	-	+	++
Tympanic border cells	+	+	++	-	-
Marginal and/or intermediate cells	++	-	-	++	++
Basal cells	+	-	-	+	+
Spiral prominence, outer sulcus, and root cells	+	+	-	-	++
Type I fibrocytes	+	-	-	++	-
Type II fibrocytes	+	-	-	-	-
Type III fibrocytes	+	-	-	-	-
Type IV fibrocytes	+	-	+	-	-
Type V fibrocytes	+	-	-	+	-
Fibrocytes in the spiral limbus	+	++	-	++	-
Interdental cells	+	+	-	-	++
Reissner’s membrane epithelial cells	+	+	+	-	+
Spiral ganglion cells	++	+	+	+	-
Vasculature	+	-	++	-	-

For LRRC8A, LRRC8C, LRRC8D, LRRC8E expression data is derived from immunofluorescent stainings. Data for LRRC8B expression is derived from X-Gal stainings. ++, intense staining; +, weak staining; -, no staining could be detected.

**Figure 2:**
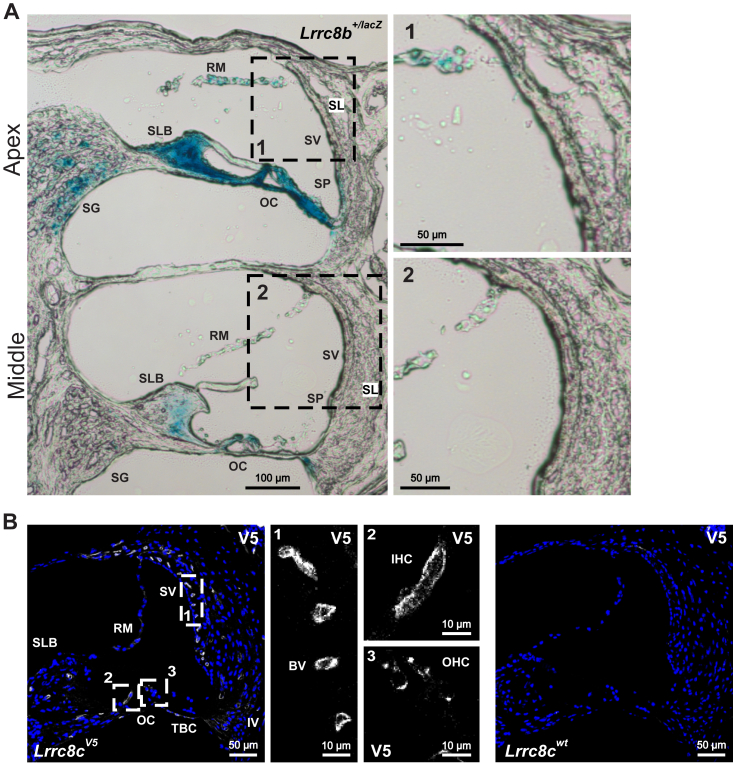
**Expression of LRRC8B and LRRC8C subunits in cochlea.***A*, X-gal stained cochlear section from 6-weeks-old *Lrrc8b*^+/^^*lacZ*^ mouse. The *Lrrc8b* promotor is active in cells of the spiral ganglion (SG), organ of Corti (OC), Reissner`s membrane (RM), spiral limbus (SLB), and spiral prominence, and outer sulcus region (SP). No X-gal staining was observed in the stria vascularis (SV) and fibrocytes in the spiral ligament (SL). *B*, LRRC8C, as detected by V5 labeling of cochlea form 12 weeks-old *Lrrc8c*^*V5/V5*^ mice, is prominently expressed in blood vessels (*e.g.* in the stria vascularis (SV) and spiral limbus (SLB)) and hair cells (IHC and OHC; OC–organ of Corti). Reissner`s membrane (RM), type IV fibrocytes (IV) and tympanic border cells (TBC) also express LRRC8C. WT mice served as a negative control. Images are collected from at least two different mice. IHC, inner hair cell; OHC, outer hair cell; X-gal, 5-bromo-4-chloro-3-indolyl-β-D-galactopyranoside.

Attempts to localize **LRRC8B** by immunohistochemistry using homozygous *Lrrc8b*^*smFPMyc*^ (smFPMyc, spaghetti monster fluorescent protein containing myc tags) KI mice (38) were unsuccessful because of a low signal to noise ratio. This suggested a low inner ear expression compared to the kidney where proximal tubules are prominently stained in *Lrrc8b*^*smFPMyc*^ KI mice (38). We therefore resorted to X-gal staining of cochleae from mice expressing lacZ under the control of the *Lrrc8b* promoter (Fig. 2*A*). This resulted in intense labeling of the OC and adjacent structures, but not of the stria vascularis (Table 1). Likewise, in the vestibular organ X-gal labeled sensory epithelia, but not in the endolymph-secreting dark cells (Fig. S3*A*).

**LRRC8C** was detected in homozygous *Lrrc8c*^*V5*^ mice (38) in which two V5-epitopes are fused to LRRC8C. Prominent labeling was seen in inner cochlear hair cells and less strong expression in outer hair cells (Fig. 2*B*). Other cells of the OC, including supporting cells, appeared negative. The blood vessels penetrating the stria vascularis were brightly stained (Fig. 2*B*). By contrast, the epithelia of the stria and most of the underlying fibrocytes lacked detectable LRRC8C-V5 labeling (Fig. 2*B*). Tympanic border cells were intensely stained (Fig. 2*B*). Weaker labeling was observed in several other cell types (Table 1). In the vestibular organ, sensory hair cells showed weak, but specific staining, and blood vessels were prominently labeled (Fig. S3*B*).

**Figure 3:**
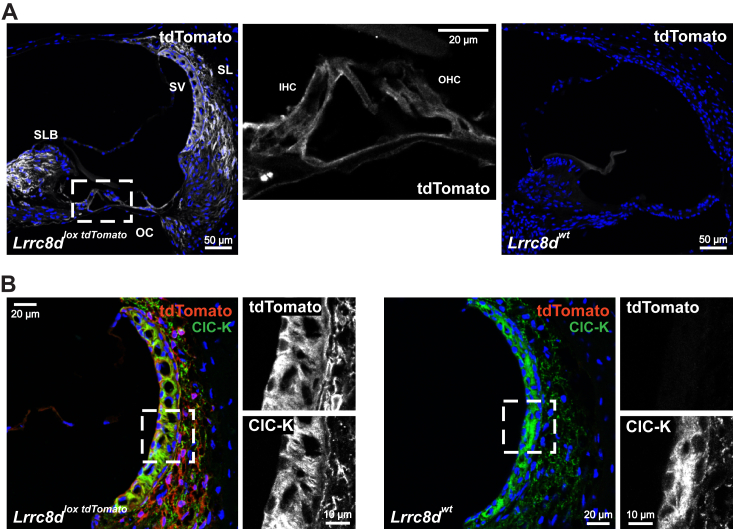
**Expression of LRRC8D subunit in cochlea.***A*, LRRC8D, detected by tdTomato staining of a cochlea from 16-weeks-old *Lrrc8c*^*lox tdTomato/lox tdTomato*^ mouse, is found in stria vascularis (SV), inner and outer hair cells (IHC and OHC) in organ of Corti (OC), and fibroblasts of spiral limbus (SLB), and spiral ligament (SL). *B*, LRRC8D expression in the lateral cochlear wall. Marginal cells of the stria vascularis are identified by staining for ClC-K Cl^-^ channels. Same mouse as in (*A*). WT mice served as negative controls. Images are collected from at least two different mice. tdTomato, tandem dimer tomato (a fluorescent protein); ClC-K, member of chloride channel family CLC.

**LRRC8D** expression, as assessed in homozygous *Lrrc8d*^*tdTomato*^^*-lox*^ mice (38, 39), differed conspicuously from those of LRRC8A, B, and C (Fig. 3, *A* and *B*). LRRC8D was prominently expressed in type I and V fibrocytes in the spiral ligament, and fibrocytes in the spiral limbus, and to lower extent in sensory hair cells (Fig. 3*A* and Table 1). Of note, LRRC8D was also detected in epithelial cells of the stria vascularis (Fig. 3*B*), where labeling resembled that for LRRC8A-HA (Figs. 1*D* and S1*B*). Staining was again compatible with either basolateral labeling of marginal cells, where it overlapped with ClC-K (Fig. 3*B*), or apical labeling of intermediate cells. No expression in apical membranes of marginal cells was observed. In the vestibular organ LRRC8D was prominently expressed in fibrocytes, but not in the sensory hair cells (Fig. S3*C*).

**Figure 4:**
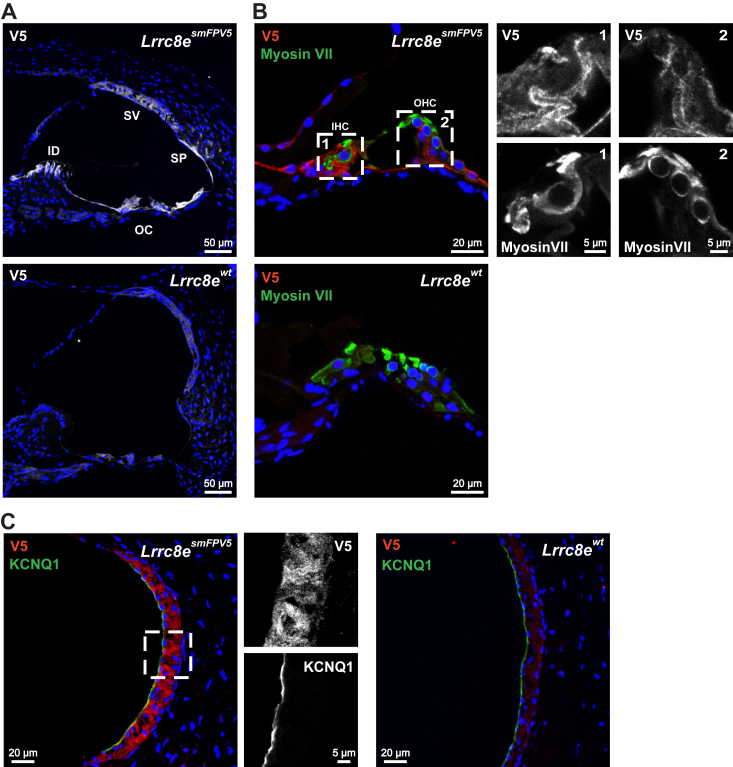
**Expression of LRRC8E subunit in cochlea.***A*, LRRC8E, detected by V5 signal 12-weeks-old *Lrrc8e*^*smFPV5/smFPV5*^ mouse, is expressed in stria vascularis (SV), spiral prominence and outer sulcus region (SP), interdental cells (ID), inner and outer hair cells (IHC and OHC) in the organ of Corti (OC), and supporting cells. *B*, LRRC8E expression in inner and outer hair cells (IHC and OHC), labeled with hair cell marker myosin VII. The 12-weeks-old *Lrrc8e*^*smFPV5/smFPV5*^ mouse. *C*, LRRC8E expression in stria vascularis. Apical membranes of marginal cells labeled with KCNQ1. 13 weeks-old *Lrrc8e*^*smFPV5/smFPV5*^ mouse. WT mice served as negative controls. Images are collected from at least two different mice.

Finally, **LRRC8E** was detected using *Lrrc8e*^*smFPV5*^ mice in which a GFP-variant carrying several V5 epitopes was fused to LRRC8E (38). LRRC8E was found in both IHCs and OHCs of the cochlea and in the stria vascularis (Fig. 4, *A*–*C*) with a labeling pattern similar to LRRC8A and LRRC8D (Table 1). Additionally, it was substantially expressed in the spiral prominence and in interdental cells (Fig. 4*A*). In the vestibular organ we could not detect LRRC8E above background.

**Figure 5:**
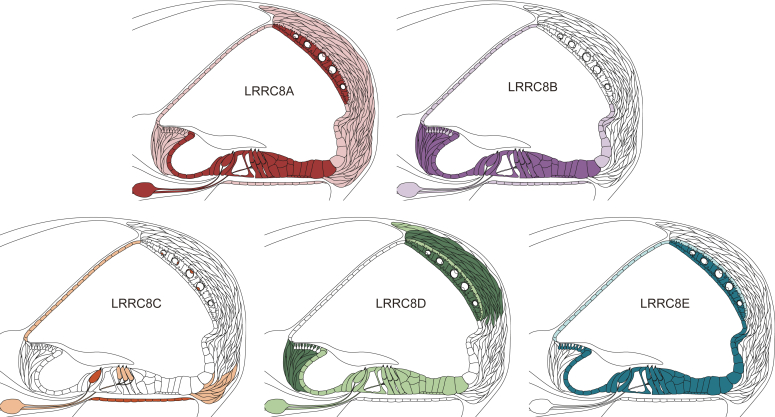
**Schematic depiction of VRAC subunit expression.** LRRC8A is broadly expressed through the entire cochlea, while nonessential VRAC subunits LRRC8B-LRRC8E display more constrained localization pattern. VRAC, volume-regulated anion channel.

In conclusion, individual VRAC subunits display remarkably different expression patterns in the cochlea (Fig. 5 and Table 1). The diversity of VRACs in the inner ear suggests that they carry out different functions.

### Disruption of *Lrrc8a* causes progressive degeneration of the OC

To determine the potential roles of VRACs in hearing, we disrupted all five *Lrrc8* genes in mice. Disruption of LRRC8A, the only essential VRAC subunit (14), completely abolishes VRAC activity, resulting in high embryonic and early postnatal lethality of *Lrrc8a*^−/−^ mice (45). We therefore crossed *Lrrc8a*^*lox/lox*^ mice (39) with SRY-box transcription factor 10 (Sox10)-Cre mice (46) we had used previously to disrupt *Bsnd* in the inner ear (44). Sox10-Cre; *Lrrc8a*^*lox/lox*^ mice (for short, Δ*Lrrc8a* mice) were viable, fertile, and lacked immediately visible phenotype. Crossing Sox10-Cre with R26R reporter mice confirmed widespread recombinase activity in cochlear and vestibular cells or their precursors (Fig. S4*A*), with the exception of fibrocytes and vascular endothelial cells (46). Likewise, HA-staining of Δ*Lrrc8a* cochleae revealed efficient deletion of LRRC8A-HA in large parts of the inner ear, including the OC and the stria vascularis, but sparing the vasculature (Fig. S4*B*). Similar results were obtained in the vestibular organ (data not shown).

**Figure 6:**
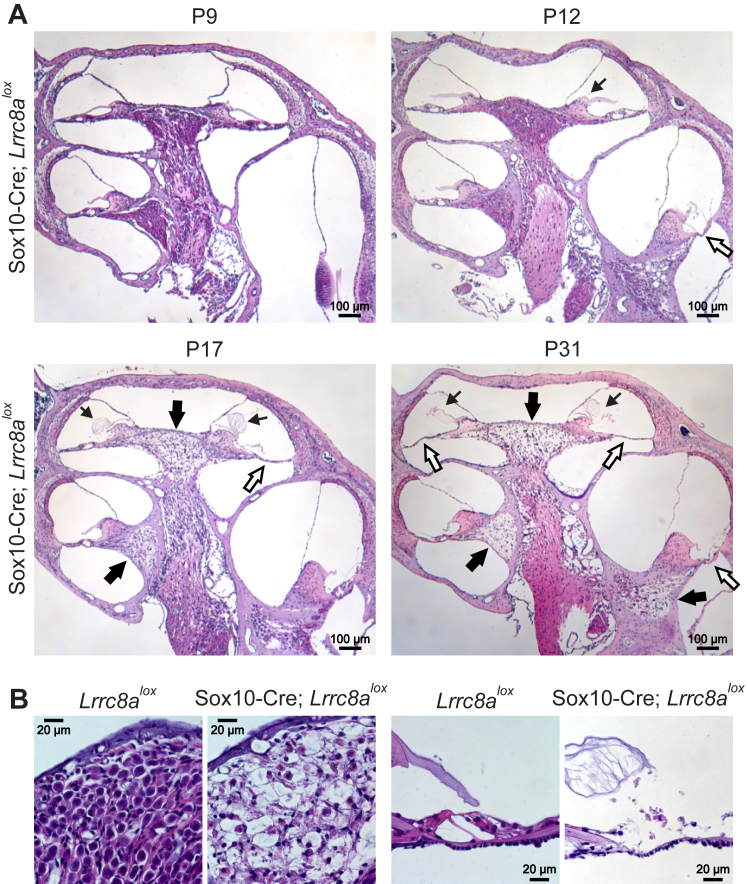
**LRRC8A ablation leads to cochlear degeneration.***A*, H&E staining of cochlear sections from Sox10-Cre; *Lrrc8a*^*lox/lox*^ mice. At P9, before the onset of hearing, cochlear anatomy is normal. Beginning with the onset of hearing (at P12–13), degeneration of spiral ganglion neurons (*thick black arrows*) and organ of Corti (*white arrows*) develops. *Thin black arrows* indicate swollen and/or detached from the spiral limbus tectorial membranes. *B*, degeneration of spiral ganglion (*left*) and organ of Corti (*right*) in 15-17-days-old Sox10-Cre; *Lrrc8a*^*lox/lox*^ mice. The tectorial membrane is swollen, and the organ of Corti and the spiral ganglion are degenerated. Images are collected from at least two different mice. Sox10, SRY-box transcription factor 10.

Until the onset of hearing, H&E staining of cochlear sections from Δ*Lrrc8a* mice revealed no morphological abnormalities. Beginning with P12, however, progressive degeneration of the OC was observed, and after P28 the OC was absent in all cochlear turns in 11 out of 18 inner ears (Fig. 6*A*). In parallel, a progressive, strong degeneration of the spiral ganglion, which innervates the OC, was observed (Fig. 6, *A* and *B*). In several mice the tectorial membrane, which normally is in contact with the stereocilia of sensory hair cells, was swollen (Fig. 6, *A* and *B*).

**Figure 7:**
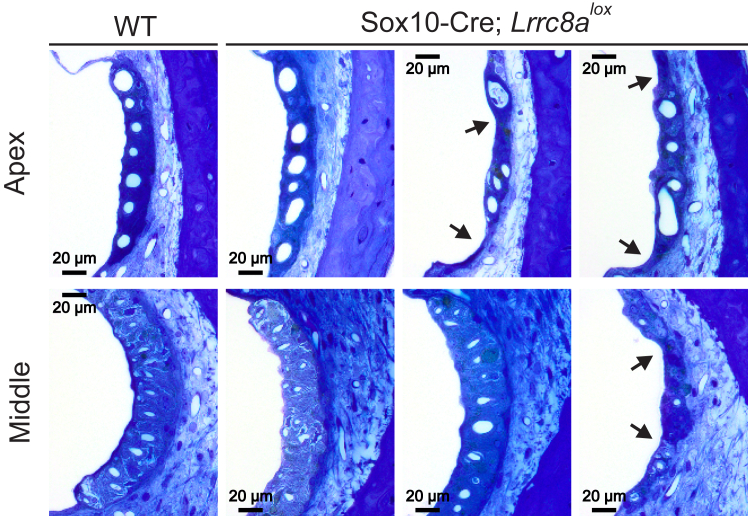
**Changes in *Lrrc8a* KO stria vascularis.** Toluidine blue-stained semithin strial sections from three 1-year-old Sox10-Cre; *Lrrc8a*^*lox/lox*^ and one WT (ctrl) mice. In two out of three investigated inner ears, the stria is markedly thinned (indicated by *arrows*). Sox10, SRY-box transcription factor 10.

**Figure 8:**
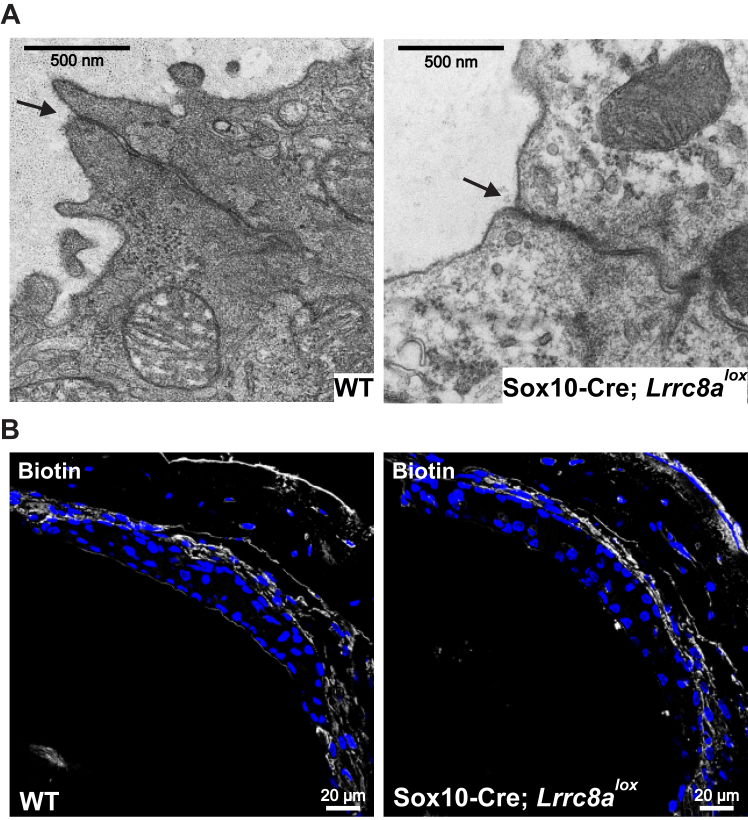
**Intact tight junctions and strial barrier in *Lrrc8a* KO stria vascularis.***A*, electron microscopy images of tight junctions (*arrows*) between strial marginal cells. Tight junctions are intact in Sox10-Cre; *Lrrc8a*^*lox/lox*^ mice. *B*, Sox10-Cre; *Lrrc8a*^*lox/lox*^ mice have intact strial barrier. Inner ears were perfused with biotin, which was later labeled with FITC-conjugated streptavidin in frozen sections. The figure depicts 3-weeks-old mice in both panels. Images are collected from at least two different mice. Sox10, SRY-box transcription factor 10.

As assessed by H&E staining, the stria vascularis appeared normal up to at least 3 months of age. It showed variable degeneration in 1 year-old mice (Fig. 7). Tight junctions between marginal cells appeared normal by electron microscopy (Fig. 8*A*), and the overall integrity of the diffusion barrier was unaffected as ascertained by biotin injections (44) (Fig. 8*B*). The normal position of Reissner’s membrane (Fig. 6*A*) indicated that K^+^-secretion by the stria was sufficiently high to keep it in place, in contrast to mouse models with severely impaired strial K^+^ secretion (47, 48, 49, 50).

### Morphological consequences of disruption of nonessential VRAC subunits

**Figure 9:**
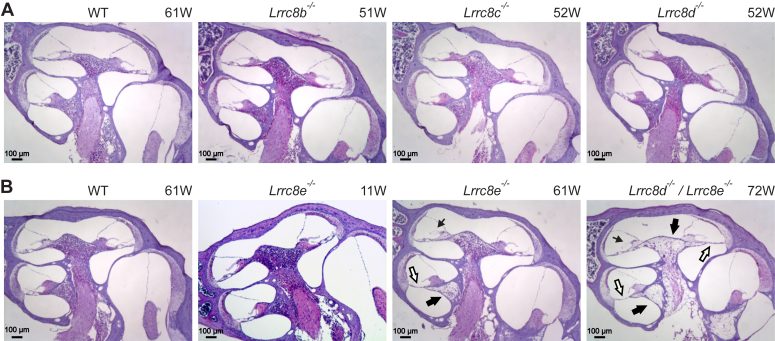
**Cochlear morphology upon disruption of nonessential single VRAC subunits.***A*, no morphological changes observed in cochleae of 1-year-old *Lrrc8b*^*−/−*^ (N = 4), *Lrrc8c*^*−/−*^ (N = 3) and *Lrrc8d*^*−/−*^ (N = 3) mice. *B*, degeneration of spiral ganglion (*thick black arrows*) and/or of organ of Corti (*white arrows*) in the middle turn is observed in 60% (7 out of 12) of investigated ears of 1-year-old *Lrrc8e*^*−/−*^ but not in 11-weeks-old animals. In 60% (7 out of 12) of studied ears, swollen tectorial membranes were found (*thin black arrow*). N = 6 for old mice, N = 5 for young mice. The 1-year-old *Lrrc8d*^*−/−*^*; Lrrc8e*^*−/−*^ displayed significantly more severe degeneration of spiral ganglion (*black arrows*) and organ of Corti (*white arrows*) and tectorial membrane swelling (*thin black arrow*) than *Lrrc8e*^*−/−*^ animals. N = 3 for *Lrrc8d*^*−/−*^*; Lrrc8e*^*−/−*^. N refers to the number of investigated KO mice. Numbers on the *top right* side of the panels refer to the animal age in weeks. VRAC, volume-regulated anion channel.

**Figure 10:**
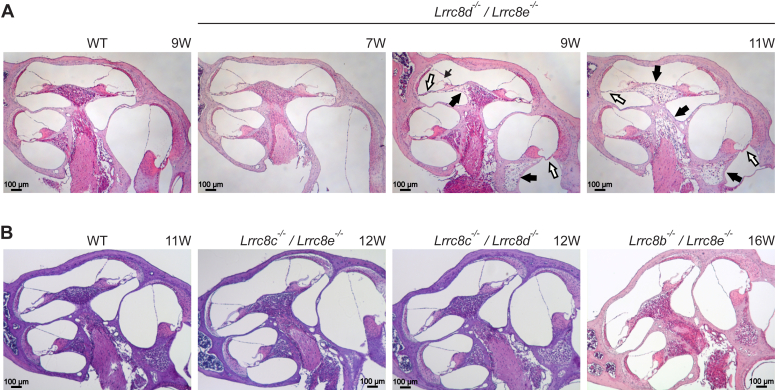
**Cochlear morphology upon disruption of two nonessential VRAC subunits.***A*, late-onset cochlear degeneration in mice constitutively lacking LRRC8D and LRRC8E. The cochlea from the 7-weeks-old mouse is either normal or displays mild degeneration, while the cochleae from the 9- and 11-weeks-old *Lrrc8d*^*−/−*^*; Lrrc8e*^*−/−*^ mice reveal degenerated spiral ganglion neurons (*thick black arrows*) and organ of Corti`s (*white arrows*). The *thin black arrow* in the image of the 9-weeks-old mouse indicates a tectorial membrane that is detached from the spiral limbus. N = 6 across all ages. *B*, no morphological changes observed in cochleae of 12-weeks-old *Lrrc8c*^*−/−*^*/Lrrc8e*^*−/−*^ (N = 2), 12-weeks-old *Lrrc8c*^*−/−*^*/Lrrc8d*^*−/−*^ (N = 4), and 16-weeks-old *Lrrc8b*^*−/−*^*/Lrrc8e*^*−/−*^ (N = 4) mice. N refers to the number of investigated KO mice. Numbers on the *top right side* of the panels refer to the animal age in weeks. VRAC, volume-regulated anion channel.

Except for *Lrrc8e*^−/−^ mice aged over 1 year, no morphological changes were observed in the inner ear when nonessential VRAC subunits were disrupted singly (in *Lrrc8b*^−/−^, *Lrrc8c*^*−/−*^, *Lrrc8d*^−/−^ and *Lrrc8e*^−/−^ mice) (Fig. 9, *A* and *B*). Only old *Lrrc8e*^−/−^ mice displayed degeneration of the OC and the spiral ganglion, but in contrast to Δ*Lrrc8a* degeneration was observed in 7 out of 12 of studied inner ears from six animals at old age (older than 1 year) (Fig. 9*B*). We also examined four strains with parallel disruption of two VRAC subunits, *Lrrc8b*^−/−^/*Lrrc8e*^−/−^, *Lrrc8c*^−/−^/*Lrrc8e*^−/−^, *Lrrc8c*^−/−^/*Lrrc8d*^−/−^, and *Lrrc8d*^−/−^/*Lrrc8e*^−/−^ mice (Fig. 10, *A* and *B*). All four double KO lines were viable and fertile. Only *Lrrc8d*^−/−^/*Lrrc8e*^−/−^ mice showed inner ear phenotypes resembling those of Δ*Lrrc8a* mice, including degeneration of the OC and the spiral ganglion. The onset of degeneration (around 9 weeks of age) was markedly slower than in Δ*Lrrc8a* mice, but considerably faster than in *Lrrc8e*^−/−^ mice (Figs. 6*A*, 9*B*, and 10*A*).

Comparison of the expression patterns of LRRC8 subunits with the pathology resulting from their ablation suggests that the degeneration of both, the OC and the spiral ganglion, are secondary to changes in the stria vascularis. Marginal and/or intermediate cells of the stria express LRRC8A, D, and E, with undetectable expression of other LRRC8 subunits (Table 1 and Fig. 5). This expression pattern agrees perfectly with the proteome obtained from rat stria vascularis (51). Since LRRC8A homomers yield virtually no currents (14), very little VRAC activity will be left in *Lrrc8d*^−/−^/*Lrrc8e*^−/−^ stria. This contrasts with hair cells in which we detected all LRRC8 subunits, and spiral ganglia with expression of LRRC8A, B, C, and D. Hence, the function of strial cells may be more severely compromised than that of hair or ganglion cells. The faster degeneration of both, the OC and the spiral ganglion, in Δ*Lrrc8a* compared to *Lrrc8d*^−/−^/*Lrrc8e*^−/−^ mice might be owed to the expression of other LRRC8 subunits in these tissues, resulting in residual VRAC activity in *Lrrc8d*^−/−^/*Lrrc8e*^−/−^ mice.

### Secondary changes in other ion transport proteins

Our results thus suggested that the degeneration of the OC and the spiral ganglion upon VRAC ablation is due to an impairment of the stria vascularis. Strial ion transport is contingent on a variety of transport proteins (2, 6) including KCNQ1/KCNE1 K^+^ channels (47, 52) and ClC-K/barttin Cl^-^ channels (44, 53). Their disruption causes deafness by directly affecting ion transport across the stria. However, gene disruption may have secondary effects on other transporters. Indeed, changes in cochlear expression of ion channels have been observed upon disruption of pendrin, an anion exchanger found in the spiral ligament, or of the BK (KCNMA1) Ca^2+^-activated K^+^ channel (54, 55).

**Figure 11:**
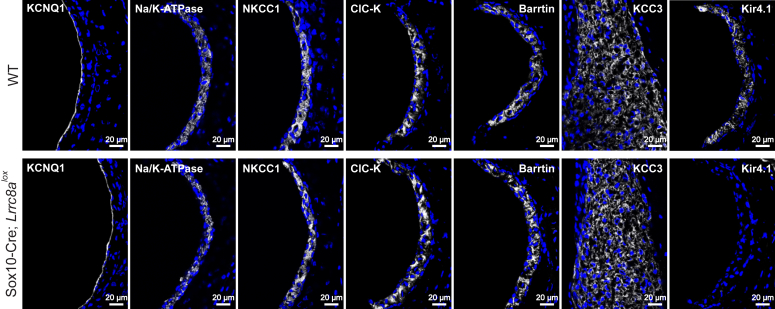
**Expression of different transporters in *Lrrc8a* KO stria vascularis and spiral ligament.** Effect of *Lrrc8a* disruption on expression levels of several ion channels and transporters in the stria vascularis and spiral ligament (KCC3) of the middle cochlear turn of 2-weeks-old Sox10-Cre; *Lrrc8a*^*lox/lox*^ mice. Strikingly, Kir4.1 cannot be detected after *Lrrc8a* disruption, whereas staining intensities for KCNQ1, Na,K-ATPase, NKCC1, ClC-K, its subunit barttin, and KCC3 are unchanged. Images are collected from at least two different mice. Sox10, SRY-box transcription factor 10.

**Figure 12:**
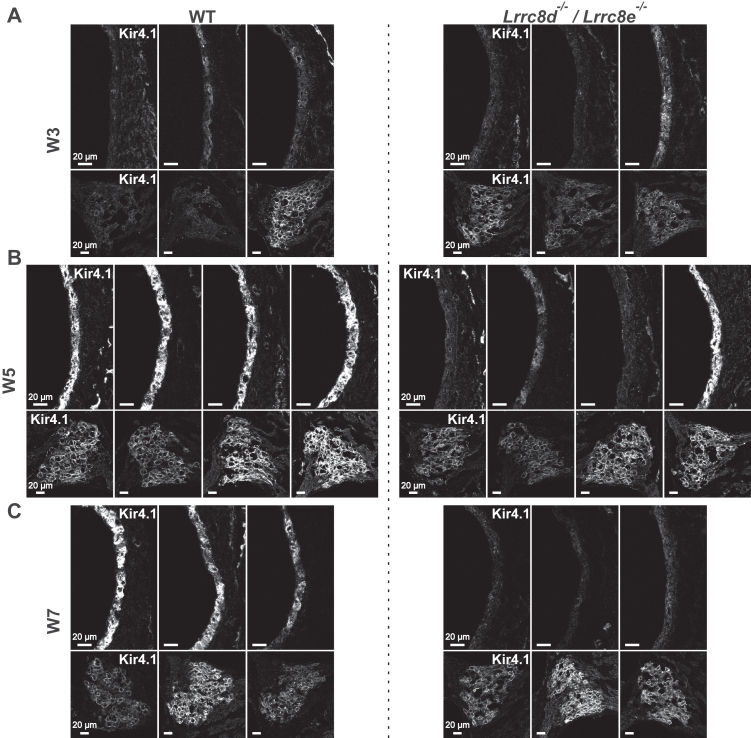
**Kir4.1 expression is significantly reduced in stria vascularis, but not in spiral ganglion of *Lrrc8d***^***−/−***^***Lrrc8e***^***−/−***^**mice above 5-weeks-old.** Stria vascularis (SV, *upper rows*) and spiral ganglion (SG, *lower rows*) of middle turn of cochlea were stained for Kir4.1 in 3- (*A*), 5- (*B*) and 7- weeks (*C*) old mice. Each pair of SV and SG images is from one mouse. N = 3 for W3 and W7, N = 4 for W5. N refers to the number of investigated mice.

Immunohistochemistry of marginal cells from two weeks-old Δ*Lrrc8a* mice showed that neither expression of apical KCNQ1 K^+^-channel, nor of basolateral ClC-K/barttin Cl^-^ channels were changed (Figs. 11 and S5*A*). Likewise, the abundance of basolateral Na^+^/K^+^-ATPase and NKCC1 (a NaK2Cl cotransporter) appeared normal. Expression of the KCl-cotransporter KCC3, whose disruption also causes deafness (56), was not changed, either. However, there was a striking downregulation of the inwardly rectifying K^+^ channel Kir4.1 (KCNJ10) of intermediate cells (Figs. 11 and S5*A*). Similar downregulation of the Kir4.1 protein was observed in *Lrrc8d*^−/−^/*Lrrc8e*^−/−^ mice, although later in time (Fig. 12, *A*–*C*). We also examined the expression of pendrin (SLC26A4), an anion exchanger expressed in the spiral ligament whose disruption secondarily decreases Kir4.1 protein levels (54). Pendrin expression was also downregulated at the protein level, albeit less than that of Kir4.1 (Fig. S5, *A* and *B*). Quantitative RT-PCR form RNA extracted from preparations containing the stria and the spiral limbus revealed unchanged *Kcnj10* and *Slc24a4* mRNA levels (Fig. S5*C*).

### Consequences of LRRC8 channel disruption on hearing and the endolymph

**Figure 13:**
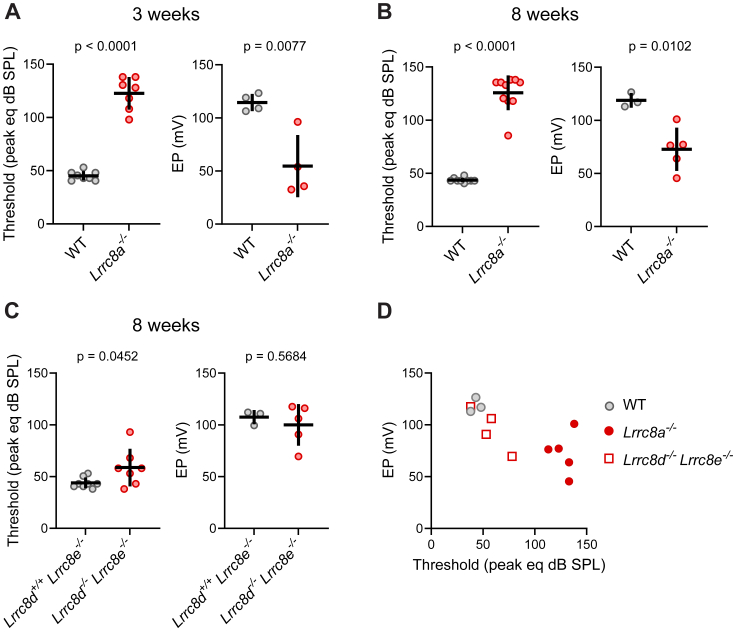
**Hearing loss in *Lrrc8a***^***−/−***^**and *Lrrc8d***^***−/−***^***/Lrrc8e***^***−/−***^**mice.***A* and *B*, *Lrrc8a*^*−/−*^ mice display a significant hearing loss already at 3 weeks old, as measured by auditory brainstem response (ABR; *left* panels), as well as a significant decrease in endocochlear potential (*right* panels). ABR: N = 8 (WT), N = 7 (KO) in 3 weeks group, N = 9 (WT), N = 10 (KO) in 8 weeks old group, EP: N = 4 (WT and KO) in 3 weeks group, N = 3 (WT), N = 5 (KO) in 8 weeks group. Mean ± SD, unpaired *t* test (ABR 3 weeks old, EP 3 and 8 weeks old), Mann-Whitney test (ABR 8 weeks old). *C*, *Lrrc8d*^*−/−*^*Lrrc8e*^*−/−*^ double KO mice display a variable tendency toward hearing loss and reduced EP at 8 weeks of age. ABR: N = 8 (*Lrrc8d*^*+/+*^*Lrrc8e*^*−/−*^), N = 7 (*Lrrc8d*^*−/−*^*Lrrc8e*^*−/−*^), EP: N= 3 (*Lrrc8d*^*+/+*^*Lrrc8e*^*−/−*^), N = 5 (*Lrrc8d*^*−/−*^*Lrrc8e*^*−/−*^). Mean ± SD, unpaired *t* test. *D*, correlation between ABR and EP values from individual ears. In *Lrrc8d*^*−/−*^*Lrrc8e*^*−/−*^ mice higher ABR is associated with lower EP. The same data as in *B* and *C*. Only data from ears that successfully underwent ABR and EP measurements are plotted. N refers to the number of investigated mice, when possible data from two ears from the same mouse was averaged. SPL, sound pressure level, eq, equivalent. ABR, auditory brainstem response; EP, endocochlear potential.

Measurements of auditory brainstem responses (ABRs) indicated that *ΔLrrc8a* mice were deaf already at 3 weeks of age (Fig. 13, *A* and *B*). While in these mice, cochlear degeneration started immediately after the onset of hearing (P12) (Fig. 6*A*), a large proportion of OCs appeared morphologically intact at that age. Since ABR reflects the output of all OCs, this suggests that apparently intact OCs are impaired functionally. As transduction currents of hair cells depend on the electrochemical driving force for K^+^ entry from the endolymph, we measured the electrical potential of the endolymph with microelectrodes (44). As expected from the secondary loss of Kir4.1 (Figs. 11 and S5*A*), which is crucial for the generation of the EP (41), EP was reduced from a normal value of + 116 ± 7 mV to + 65 ± 25 mV in both 3- and 8 weeks-old mice (Fig. 13, *A* and *B*). This reduction is probably sufficient to cause the observed severe hearing loss by markedly reducing transduction currents of both IHCs and OHCs. While IHCs directly convey sensory output to the brain, OHCs need the transduction currents to drive the motor protein prestin that mechanically amplifies the vibrations associated with sound. Since selective loss of this “cochlear amplifier” as in *Kcnq4*^−/−^ mice (57) already causes a hearing loss of about 60 dB, reduced EP, by affecting both IHC and OHC currents, markedly reduces hearing sensitivity. Reduction of the EP may secondarily lead to the observed slow degeneration of sensory hair cells and OCs as observed in other mice with severely decreased EP (44).

Compared to Δ*Lrrc8a* mice, *Lrrc8d*^*−/−*^ *Lrrc8e*^*−/−*^ mice displayed a much milder and variable hearing loss at 8 weeks of age (Fig. 13*C*). This correlates with their slower degeneration of the OC which begins at around the same age (Fig. 10*A*). It also agrees with an only slight and overall statistically not significant reduction in the EP (Fig. 13*C*). Nonetheless, when comparing ABR and EP in individual mice, there was a correlation between reduction of EP and increase in hearing threshold as seen much more clearly with Δ*Lrrc8a* mice (Fig. 13*D*).

With the anecdotal exception of one old Δ*Lrrc8a* mouse, which showed spontaneous head tilting, no obvious consequences of the variable vestibular organ degeneration were observed. However, more sensitive tests are required to detect more subtle effects on motor coordination.

## Discussion

Although volume-regulated VRAC/LRRC8 channels are widely distributed, individual LRRC8 subunits have highly distinct expression patterns, as found here for the inner ear. We discovered that deletion of either the essential VRAC subunit LRRC8A, or combined disruption of LRRC8D and E, led to a degeneration of the OC and other inner ear structures. VRAC-mediated cell volume regulation was believed to be crucial for fundamental processes such as cell division, growth, and migration (9, 58). After VRAC’s molecular identification (14), however, genetic disruption of *Lrrc8* genes revealed that VRACs are dispensable for the survival and growth of individual cells and for the overall development of the organism. The alterations in cochlear morphology and function observed here also appear unrelated to impaired cell growth and survival or defective cell volume regulation. Cochlear degeneration rather appears to be an indirect consequence of VRAC disruption in the stria vascularis as revealed by a marked decrease in EP. This decrease could be attributed to a downregulation of the strial Kir4.1 potassium channel. We hypothesize that this downregulation results from impaired VRAC-dependent transport of organic molecules, possibly glutathione, which provide protection against metabolic and oxidative stress.

### Primary role of the stria in the degeneration of various inner ear structures

The similar inner ear phenotypes of Δ*Lrrc8a* and *Lrrc8d*^−/−^/*Lrrc8e*^−/−^ mice pointed to the stria (which expresses LRRC8A, D, and E, but almost no other LRRC8 isoform) as primary actor in the degeneration of the OC and other inner ear structures. Indeed, we found that the EP, which is generated by the stria, was markedly reduced upon VRAC ablation. We excluded that this reduction was owed to an unspecific leakiness of the stria. It was neither caused by strial degeneration which occasionally occurred at old ages.

**Figure 14:**
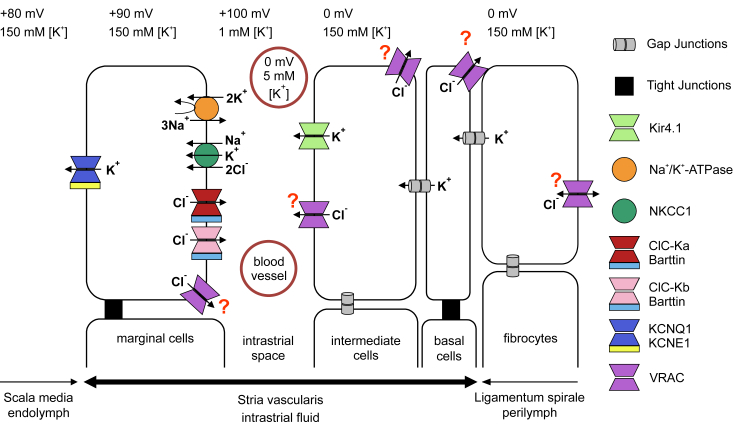
**Scheme of endocochlear potential generation**. Potassium is secreted by KCNQ1/KCNE1 potassium channels in the apical membranes of marginal cells. This secretion requires K^+^ accumulation into marginal cells across their basolateral membrane, a process carried out by the Na^+^K^+^-ATPase and the Na^+^-gradient driven Na^+^K^+^2Cl^-^ NKCC1 cotransporter. Chloride taken up by NKCC1 needs to be recycled over the basolateral membrane of marginal cells by Cl^-^ channels, most prominently by ClC-Ka and ClC-Kb channels associated with their obligatory subunit barttin. VRAC/LRRC8 channels in marginal cells may play an additional, probably minor role in this recycling process. The positive endocochlear potential depends critically on Kir4.1 (KCNJ10) in the apical membrane of intermediate cells. They generate a large positive K^+^-diffusion potential in the cleft between intermediate and marginal cells. Kir4.1 is downregulated in the absence of the obligatory VRAC subunit LRRC8A, or when both LRRC8D and LRRC8E are missing. This downregulation may be related to VRAC’s ability to transport organic molecules such as glutathione, as suggested by the sensitivity of Kir4.1 protein levels to oxidative stress. VRAC may perform this role in several cell types because it is expressed in all stria vascularis cell types (LRRC8A, D and E) and fibrocytes (LRRC8A and D for fibrocytes I). Figure adapted from (44). VRAC, volume-regulated anion channel.

Strial marginal cells secrete potassium into the endolymph through apical KCNQ1/KCNE1 K^+^ channels (2, 47, 52). Potassium secretion relies on cytoplasmic K^+^ accumulation by the basolateral Na,K-ATPase and the NaK2Cl cotransporter NKCC1, which in turn is driven by the Na^+^-gradient generated by the ATPase. Chloride is recycled across the basolateral membrane through constitutively open ClC-Ka/barttin and ClC-Kb/barttin Cl^-^ channels (44, 53) (Fig. 14). Immunolabeling of LRRC8A, D, and E is compatible with the localization of all three subunits in marginal and intermediate cells of the stria. Basolateral localization of VRAC in marginal cells would fit to the targeting of LRRC8A, B, and D to basolateral, but not apical, membranes of renal tubules (38). VRAC may provide a minor, regulated pathway for Cl^-^ recycling across basolateral membrane of marginal cells, in parallel to the larger, probably unregulated ClC-K/barttin conductance (Fig. 14). Compatible with redundant roles of these two Cl^-^ channels in K^+^ secretion, neither disruption of ClC-K/barttin (44), nor disruption of VRAC (this work) entail a collapse of Reissner’s membrane, suggesting that K^+^ secretion is maintained at levels still sufficient to sustain osmotic pressure in the scala media. Importantly, *Bsnd*^−/−^ mice, in which disruption of the essential barttin subunit (53) completely abolishes Cl^-^ currents through both ClC-K isoforms, also display severely reduced EP (44). These considerations suggest that ablation of ClC-K/barttin or LRRC8A might reduce EP in a similar manner. However, our results rather indicate fundamentally different mechanism for EP reduction upon VRAC disruption.

### Pivotal role of secondary downregulation of Kir4.1 (KCNJ10) K^+^ channel in VRAC-related deafness

A highly relevant finding was the strong downregulation of Kir4.1 (KCNJ10) in Δ*Lrrc8a* mice and in older *Lrrc8d*^−/−^/*Lrrc8e*^−/−^ mice. This inwardly rectifying K^+^ channel, found in apical membranes of intermediate cells (59), is necessary to generate the positive EP (6, 40, 41, 42, 50, 54, 60). Complete Kir4.1 ablation also reduces the K^+^ concentration of the endolymph by about 50% (42) and thereby induces a collapse of Reissner’s membrane (42, 50). The normal position of this membrane in Δ*Lrrc8a* mice indicates that VRAC KO does not completely abolish Kir4.1 expression. Of note, like Δ*Lrrc8a* mice, *Kcnj10*^−/−^ mice display degeneration of the OC and the spiral ganglion (42, 50). They also display swollen tectorial membranes (50) similar to several Δ*Lrrc8a* mice. Swollen and disorganized tectorial membrane are also seen in mice lacking pendrin (Slc26a4) (61), which display a secondary loss of Kir4.1 (54). Tectorial membranes are acellular polyelectrolyte gels which can respond to changes in cation concentrations with swelling (62). Hence, it is likely that this peculiar phenotype results from changed ion composition in the scala media as a consequence of the marked loss of Kir4.1. Most inner ear symptoms of Δ*Lrrc8a* mice might be caused by Kir4.1 downregulation.

By contrast, Kir4.1 cannot be invoked in ClC-K/barttin related deafness because strial Kir4.1 expression is unchanged in *Bsnd*^−/−^ mice (44). The decreased EP of *Bsnd*^−/−^ mice is likely caused by the lack of continuous ClC-K/barttin-mediated depolarizing Cl^-^ efflux across the basolateral membrane of the marginal cells (44) which normally contributes to the transcellular endolymph-positive potential. Although we cannot rule out a minor role of such a mechanism also in the present case, the case for a role of Kir4.1 downregulation in EP reduction in Δ*Lrrc8a* mice is compelling.

### Potential mechanism of Kir4.1 downregulation

Strial Kir4.1 protein levels were reduced upon VRAC disruption without changes in *Kcnj10* transcript levels, suggesting an effect on protein stability. LRRC8 and KCNJ10 channels are coexpressed in intermediate cells, but it seems unlikely that direct VRAC/Kir4.1 protein interaction is needed for Kir4.1 stability. Intriguingly, a strong reduction of strial Kir4.1 protein levels has been observed in *Slc26a4*^−/−^ mice which lack the anion exchanger pendrin (54). Because Kir4.1 and pendrin are expressed in different cell types (pendrin is found mainly in the spiral prominence), downregulation of Kir4.1 by *Slc26a4*^−/−^ disruption must be indirect. Increased levels of oxidized and nitrated proteins in cochleae of *Slc26a4*^−/−^ mice indicate that loss of Kir4.1 is caused by increased oxidative and free radical stress (63), which might be related to an acidification of the endolymph without bicarbonate-transporting pendrin (54). Free radical stress reduces Kir4.1 protein levels also in heterologous expression (63). Although the moderate reduction of pendrin levels in Δ*Lrrc8a* mice, by whatever mechanism, might contribute to a loss of Kir4.1, such a contribution must be minor because heterozygous *Slc26a4*^*+*/−^ mice display no phenotype.

Interestingly, VRACs can transport glutathione (32, 33), a tripeptide which protects against oxidative stress. The stria contains high levels of glutathione (64) and expresses various isoforms of glutathione transferases (65). VRAC activity is modulated by oxidative stress (27, 28, 66), with LRRC8A/C and LRRC8A/D channels being inhibited, and LRRC8A/E strongly stimulated, by oxidation (28, 66). Since both D and E isoforms are expressed in stria, it is difficult to predict whether oxidation will increase or decrease VRACs’ transport activity in this tissue. Both LRRC8D and LRRC8E are known to enable transport organic substrates through VRAC (10, 30, 31, 34), but their specific roles in glutathione transport remains unknown. In any case, VRACs ability to transport glutathione and its regulation by oxidation suggests intriguing feedback loops for the regulation of cochlear redox potentials.

### Comparison to other mouse models with reduced EP

An obvious question is which phenotypes of *Kcnj10*, *Bsnd*, and *Lrrc8a* KO mice can be attributed to their reduced EP. A complicating factor is that endolymph K^+^ concentration is halved in *Kcnj10*^−/−^ (42, 50), but not in *Bsnd*^−/−^ (44) and probably not in Δ*Lrrc8a* mice. Less positive EP, potentiated by a reduction of endolymphatic [K^+^] in *Kcnj10*^−/−^ mice, reduces the driving force for K^+^ entry through transduction channels and is sufficient to cause deafness. Similar to Δ*Lrrc8a* mice, *Bsnd*^−/−^ mice display a ∼60 dB hearing loss already at 2 weeks (44) when hearing normally starts. Degeneration of *Bsnd*^−/−^ hair cells begins only later, similar to Δ*Lrrc8a* or *Kcnj10*^−/−^ mice (50). Increased Ca^2+^ influx through transduction channels may contribute to slow hair cell degeneration in all three mouse models because of a likely increase in endolymphatic Ca^2+^ concentration. The normally low endolymph [Ca^2+^] (∼23 μM) might, in principle, be established just by passive equilibration with normal extracellular [Ca^2+^] (∼2 mM) with positive EP.

While these effects are similar in all three mouse models, there are also notable differences. Cochlear degeneration of Sox10-Cre; *Bsnd*^*lox/lox*^ mice was milder than in Sox10-Cre; *Lrrc8a*^*HA-lox/HA*^^*-lox*^ mice or in *Kcnj10*^−/−^ mice. No degeneration was observed in the spiral ganglion of *Bsnd*^−/−^ mice, which display degeneration of outer, but not IHCs. Hence, the severe degeneration of the OC and of the spiral ganglion of *Lrrc8a* mouse models cannot be attributed exclusively to a loss of the EP. Other cell-intrinsic effects such as a loss of cell volume regulation may contribute to cell damage.

Degeneration of the spiral ganglion, which innervates sensory hair cells, may be in part secondary to the severe degeneration of the OC in the present mouse model. Indeed, experimental ablation of inner and outer cochlear hair cells entails progressive degeneration of ganglion neurons (67). By contrast, no degeneration of the ganglion was reported in genetic mouse models with rather selective degeneration of OHCs (5, 57), including *Bsnd*^−/−^ mice (44). It is unclear whether this difference is owed to the sparser innervation of OHCs.

## Conclusion

VRAC is essential for hearing. Surprisingly, the deafness caused by VRAC disruption is not caused by cell loss resulting from impaired cell volume regulation. It rather results from an effect on the expression of another channel, Kir4.1, which is crucial for the generation of the EP. Collapse of the EP suffices to cause early severe hearing loss which later becomes irreversible by the degeneration of the OC and spiral ganglia. We hypothesize that the downregulation of Kir4.1 is caused by the lack of a secreted factor, possibly glutathione that is normally transported through VRAC. This would be in line with an increasing number of reports showing the physiological importance of VRAC-mediated release of molecules as diverse as glutamate (37), GABA (68, 69), ATP (69) and cGAMP (34, 35). The wide expression pattern of VRAC in the inner ear, and the ability of VRACs to transport cisplatin (10), suggests that VRAC may also play a role in ototoxicity. *Lrrc8d*^−/−^ mice (38), which lack the LRRC8D subunit which fosters cisplatin transport (10), offer the possibility to explore this hypothesis.

## Experimental procedures

### Mice

The following mouse models were generated in our laboratory: *Lrrc8a*^*lox/lox*^ (43), *Lrrc8a*^*HA*^ (43), L*rc8d*^*tdTomato*^ (39), *Lrrc8b*^*smFPMyclox*^, *Lrrc8c*^V5^, *Lrrc8e*^*smFPV5*^, *Lrrc8b*^−/−^, *Lrrc8c*^−/−^, *Lrrc8d*^−/−^ (38), and *Lrrc8e*^−/−^ (34). Sox10-Cre mice (46) were generously provided by Prof. W. D. Richardson. Mice were kept in the MDC animal facility or ZTL, Hannover Medical School. All animal experiments were approved by the respective authorities (LAGeSo (Berlin) and LAVES (Oldenburg)).

For morphological analysis, anaesthetized mice were perfused with PBS + 0.01% heparin for 2 min followed by 3 min of perfusion with fixative solution (for H&E and X-gal stainings PBS + 4% paraformaldehyde (PFA); for immunofluorescence PBS + 1% PFA; for electron microscopy, 4% PFA with 2.5% glutaraldehyde). Inner ears were collected and dissected in ice-cold PBS.

### Auditory brainstem recordings and measurements of the endocochlear potential

Auditory click-evoked brainstem responses (ABRs) were used to determine hearing thresholds in anesthetized (xylazine hydrochloride 16 mg/kg body weight and ketamin hydrochloride 120 mg/kg body weight) animals at 3 and 8 weeks of age. Alternating acoustic stimuli, covering the hearing range up to 12 kHz were applied monaurally at a rate of 21/s. Bioelectric responses were averaged 200 to 2000 times. Stimulus intensities were varied up or down, starting from 118 dB peak equivalent sound pressure level [pe dB SPL] in increments of 20 dB except near threshold where 5 dB steps were used. Thresholds as defined by the lowest level to generate a reproducible ABR wave were determined visually.

The day after ABR measurements the EP was determined in the anesthetized mice (14 mg/kg xylazine hydrochloride, 80 mg/kg ketamine hydrochloride) on a heated surgical table. The bulla was exposed and opened caudal laterally, leaving the tympanic membrane intact. Subsequently, the bone over the first turn of the cochlea, inferior to the stapedial artery, was thinned and opened. A single barreled microelectrode was inserted into scala media through the stria vascularis, and the EP was measured against a reference electrode below the skin in the neck of the animal.

### H&E staining

After perfusion inner ears were post fixed in 4% PFA in PBS at 4 °C overnight followed by decalcification with 10% EDTA + 0.02% NaN_3_ in PBS for 2 weeks at 4 °C. Then inner ears were dehydrated with ascending isopropanol series (50%, 60%, 75%, and 100% isopropanol in PBS for at least 2 h each), incubated at 60 °C first in 1:1 mixture of isopropanol with paraffin (overnight) and then in pure paraffin (6 h), followed by paraffin embedding. Paraffin blocks were cut into 5 μm sections for further staining.

For H&E staining, inner ear sections were first incubated twice in Roti-Histol (Roth, 6640.1) for 5 minutes each and then hydrated in descending ethanol series (100%, 96%, 80%, and 70% EtOH in dH_2_O for 5 min each). Sections were then incubated in dH_2_O for 2 min and stained with Mayer's hemalum solution (Sigma-Aldrich, 109249) for 6 min. Next, slides were washed with warm water for 8 min, briefly dipped in 0.25% concentrated (37%) HCl in 70% EtOH mix followed by 10 min wash with water. Sections were then stained for 2 to 3 min with eosin G solution (0.3% eosin G, five drops of acetic acid per 100 ml of solution), rinsed with distilled water and dehydrated in ascending ethanol series (70%, 80%, 96%, and 100% EtOH for 2 min each). Finally, sections were rinsed twice for 2 min in Roti-Histol and mounted in Roti-Histokitt (Roth, 6638.1). Images were taken with an AxioCam MRc5 camera (Zeiss) on an Axiophat microscope (Zeiss) with an Achrostigmat 5×/0.12, Plan-NEOFLUAR 10×/0.30, Plan-NEOFLUAR 20×/0.50, or Plan-NEOFLUAR 40×/0.75 using ZEN3.8 (ZEN lite) (Zeiss) software version 3.8.1.

### X-gal staining

Dissected inner ears were postfixed in 2 mM MgCl_2_ + 2.5% PFA + 0.2% glutaraldehyde in PBS for 10 min. Inner ears slices were then permeabilized by three times washing in 2 mM MgCl_2_ + 0.2% NP40 in PBS for 10 min and then stained with X-gal solution (2 mM MgCl_2_ + 5 mM K_3_Fe(CN)_6_ + 5 mM K_4_Fe(CN)_6_ + 1 mg/ml X-gal + 0.2% NP40 + 0.1% Na-desoxycholate in PBS) overnight at 37 °C. Inner ears were then thoroughly washed with 2 mM MgCl_2_ in PBS, fixed with 4% PFA in PBS for 10 min, and decalcified with 0.25 M EDTA + 15% sucrose + 0.02% NaN_3_ in PBS for 2 weeks at 4 °C. Afterward, inner ears were fixed with 4% PFA in PBS for 5 min and equilibrated in Tissue-Tek O.C.T. Compound (Sakura, 4583) for 2 days followed with the embedding in the same compound. A total of 12 μm thin cryosections were produced and imaged with an Axiopath microscope described in H&E staining section.

### Immunofluorescent staining

Dissected inner ears were postfixed with 1% PFA in PBS for 3 h, decalcified with 10% EDTA + 0.02% NaN_3_ in PBS for 2 weeks, and incubated in 30% sucrose in PBS overnight at 4 °C. Further, inner ears were incubated for 6 h in 1:1 mixture of 30% sucrose in PBS with Tissue-Tek O.C.T. Compound at room temperature and embedded in Tissue-Tek O.C.T. Compound. Subsequently, 8 μm thin cryosections were produced with CryoStar NX70 cryostat (Thermo Fisher Scientific).

Cryosections were fixed with 1% PFA + 0.1% Na-desoxycholate + 0.2% NP40 in PBS for 10 min and then blocked with 2% bovine serum albumin (BSA) + 3% normal goat serum + 0.5% NP40 in PBS for 1 h at room temperature. Sections were then stained overnight at 4 °C with primary antibody in 2% BSA + 1.5% normal goat serum + 0.5% NP40 followed by secondary antibody staining in the same solution for 1 h at room temperature. Finally, sections were mounted with Fluoromount-G (SouthernBiotech, 0100–01) or Epredia Shandon Immu-Mount (Thermo Fisher Scientific, 9990402), and images were acquired with the Zeiss LSM 880 confocal microscope (Axio Observer Z1 stand) with 20×/0.8, 40×/1.2, and 63×/1.4 objectives operated with ZEN2/1 SP3 (Version 14.0.28.201) (Zeiss) software.

Following primary antibodies were used: guinea pig anti-barttin (53) (1:300); rabbit anti-HA (Cell Signaling Technology, 3724; 1:200); rat anti-HA (Roche, 11867423001; 1:250); rabbit anti-myosin VIIa (*Proteus*, 25-6790; 1:1000); guinea pig anti-ClC-K (44) (1:100); rabbit anti-KCNQ1 (70) (1:500); guinea pig anti-KCNQ4 ((71), 1:200); rat anti-V5 (Biozol, Orb256446; 1:200); rabbit anti-RFP (Rockland, 600–401–379; 1:1000); chicken anti-Na,K-ATPase (Abcam, ab353; 1:100); rabbit anti-NKCC1 (gift from C. Hübner, 1:300); rabbit anti-KCC3 ((56), 1:400); guinea pig anti-Kir4.1 (gift from S. Takeuchi; 1:300); guinea pig anti-prestin (Santa Cruz, sc-22692; 1:1000). Whenever possible, staining of a similar section from mice not expressing the tag was performed to verify the antibody specificity.

For tracer permeability assays, inner ears were collected from 3-weeks-old mice and dissected in cold PBS supplemented with 1 mM CaCl_2_ (removed middle ear bones, opened round and oval windows). The cochleae were perfused from the round to the oval window with 500 μl of EZ-LinkTM Sulfo-NHS-LC-Biotin (10 mg/ml; Thermo Fisher Scientific, 21335) in PBS + 1 mM CaCl_2_ for 5 min followed by just 1 mM CaCl_2_ in PBS for 5 min. Then a hole in the bone was made at the apical turn of the cochlea, and inner ears were fixed in 4% PFA in PBS at 4 °C overnight and again for 1 h 2 days later. Further the inner ears were processed similarly to the samples for the immunohistochemistry staining. Cryosections were blocked with 2% BSA + 0.5% NP40 in PBS for 45 min and incubated with FITC-conjugated streptavidin (1:1000; Thermo Fisher Scientific, S11223) in PBS supplemented with 2% BSA and 0.5% NP40. The sections were mounted with Fluoromount-G and imaged similarly to immunohistochemistry samples.

### Transmission electron microscopy

Anaesthetized 3-weeks-old mice were perfused with 4% PFA with 2.5% glutaraldehyde in PBS for 5 min. Inner ears were dissected and post fixed in the same solution overnight at 4 °C and decalcified with 10% EDTA + 0.02% NaN_3_ in PBS for 6 days at 4 °C. Cochlea was then separated from the vestibular organ and cut in two halves. Cochleae were washed with 0.1 M cacodylate buffer and osmicated with 1% osmic acid + 1.5% hexacyanoferrate at 4 °C for 1 h followed by incubation in 1% uranyl acetate for 1 h. Cochleae were then dehydrated in methanol series (30%, 50%, 70%, and 90% MetOH for 15 min each, 100% MetOH 3 times for 20 min) and then incubated in propylene oxide (PO) for 5 min, in PO + epoxy resin (2:1) for 1 h, in PO + epoxy resin (1:1) for 1 h, in PO + epoxy resin (1:3) for 1 h, in pure epoxy resin for 1 h, in pure epoxy resin overnight, and again in pure epoxy resin for 1 h. Finally, cochlea halves were embedded in epoxy resin, which then was polymerized at 60 °C. Ultrathin 70 nm sections were produced, placed on a coarse-meshed metal grid and imaged with Zeiss 900 transmission electron microscope (Morada G2 digital camera).

### Toluidine blue staining

Cochleae of 1-year-old mice were collected and processed as described for electron microscopy. Subsequently, 1 μm semithin sections were produced, placed on a glass objective with a drop of dH_2_O, and heated to 60 °C until water got evaporated. Toluidine blue was added to the section for 20 s, and then the slides were washed and dried. The sections were mounted with Immersol 518F (Zeiss), coverslips were fixed with nail varnish, and imaged with Axiophot microscope (Zeiss) as described for H&E staining.

### Quantitative RT-PCR

Inner ears were collected from 1-year-old mice, middle ear and temporal bones were removed. Stria vascularis and spiral ligament were separated from the spiral ganglion, spiral limbus, and OC and pooled with the same part of the second cochlea from one mouse. RNA was extracted using the NucleoSpin RNA kit (Macherey-Nagel, 740955.50) following the manufacturer`s instructions. Samples were lysed by vigorous vortexing. In the end, the RNA concentration was measured using a ND-1000 spectrophotometer (NanoDrop Technologies). Afterward, 500 ng RNA of each sample was used for the complementary DNA (cDNA) synthesis. The reverse transcription of the RNA into cDNA was achieved by using SuperScript II Reverse Transcriptase (Thermo Fisher Scientific, 18064014), dNTPs (Rapidozym, GEN-011-M10), and random primers (Invitrogen, 48190011). For the quantitative PCR, the PCR reaction mix contained cDNA, 300 nM forward and reverse primer, and Power SYBR Green PCR Master Mix (Applied Biosystems, 4367659). The PCR reaction mix was run on a StepOnePlus Real-Time PCR System (Applied Biosystems). Using the ΔΔCT method, relative mRNA expression levels were calculated and normalized to mean values of the reference genes ubiquitin C, β-actin, and GAPDH. Following primers were used: *Ubc:* AGCCCAGTGTTACCACCAAG, ACCCAAGAACAAGCACAAG; *Actb:* TGTGATGGTGGGAATGGGTCAGAA, TGTGGTGCCAGATCTTCTCCATGT; *Gapdh:* TGGCAACAATCTCCACTTTGC, AGCCTCGTCCCGTAGACAAAA; *Kcnj10:* TGGTGTGGTGTGGTATCTGG, TGAAGCAGTTTGCCTGTCAC; *Slc26a4:* TCTGATGGAGGCAGAGATGA, GGCCAGCCTAACAGAGACAG; *Kcnq1:* TTTGTTCATCCCCATCTCAG, GTTGCTGGGTAGGAAGAG; *Kcnq4:* CCCGGAAACCCTTCTGTGTC, AAAGATGAGCACCAGGAACC.

### Statistical analysis

For immunohistochemistry signal quantifications, regions of interests were drawn manually around stria vascularis or spiral ligament and used to quantify mean gray value. Image quantification was done in Fiji. Corresponding N numbers and statistical tests are described in figure legends. Appropriate statistical tests were selected based on normality of data distribution (Shapiro–Wilk test). All performed t tests and Mann–Whitney tests were two-tailed.

## Data availability

All primary data are available from the corresponding author upon reasonable request.

## Supporting information

This article contains supporting information, which includes a reference to (72).

## Conflict of interest

The authors declare that they have no conflicts of interest with the contents of this article.
