# Supplementary material for: LRRC8/VRAC volume-regulated anion channels are crucial for hearing
Source: J Biol Chem. 2024 Jun 4;300(7):107436. doi: 10.1016/j.jbc.2024.107436 (PMC11260850; doi:10.1016/j.jbc.2024.107436)
Supplement: Supporting information [file mmc1.pdf]

Supporting Information

LRRC8/VRAC volume-regulated anion channels are crucial for hearing

Deborah Knecht, Mariia Zeziulia, Mit Bhavsar, Dmytro Puchkov, Hannes Maier, Thomas J. Jentsch

Figures S1 – S5

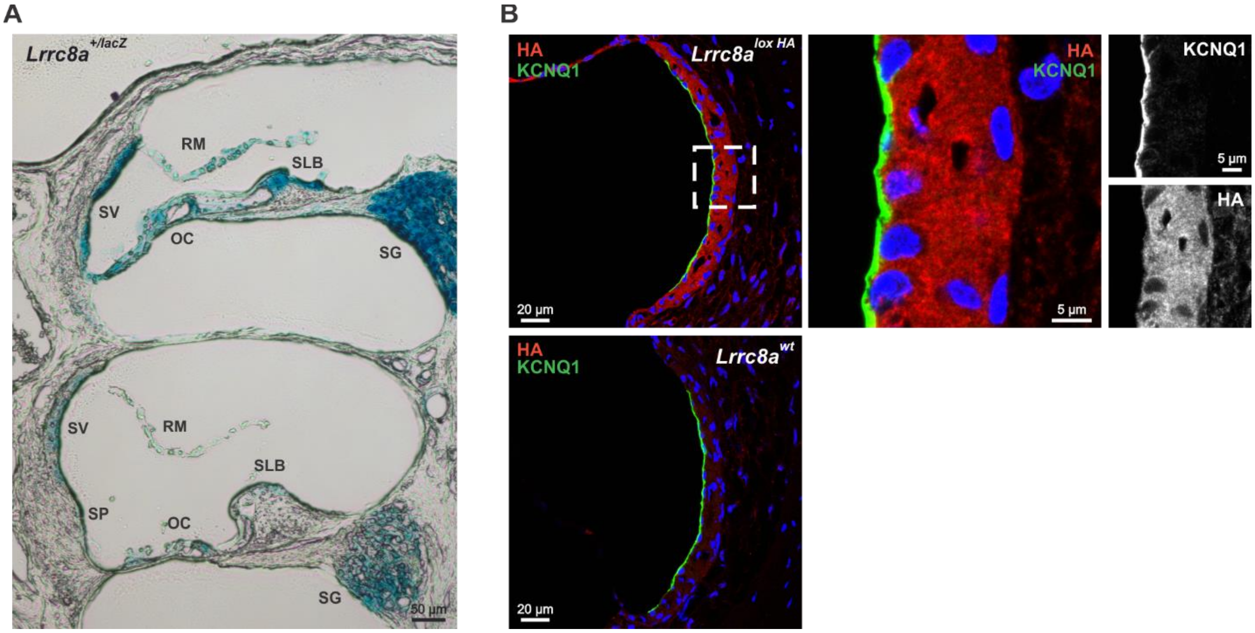

Figure S1. LRRC8A subunit expression in cochlea. A. X-Gal stained cochlear section from 13-week-old *Lrrc8a*$^{+/lacZ}$ mouse. The *Lrrc8a* promotor is active in cells of the spiral ganglion (SG), organ of Corti (OC), Reissner`s membrane (RM), stria vascularis (SV), spiral prominence and outer sulcus region (SP), and in the spiral limbus (SLB). No labeling was detected in fibrocytes and bone. **B.** LRRC8A is expressed in strial cells, labelled with marginal cell marker KCNQ1. 23-week-old *Lrrc8a*$^{lox\ HA/lox\ HA}$ mouse. Images are collected from at least 2 different mice.

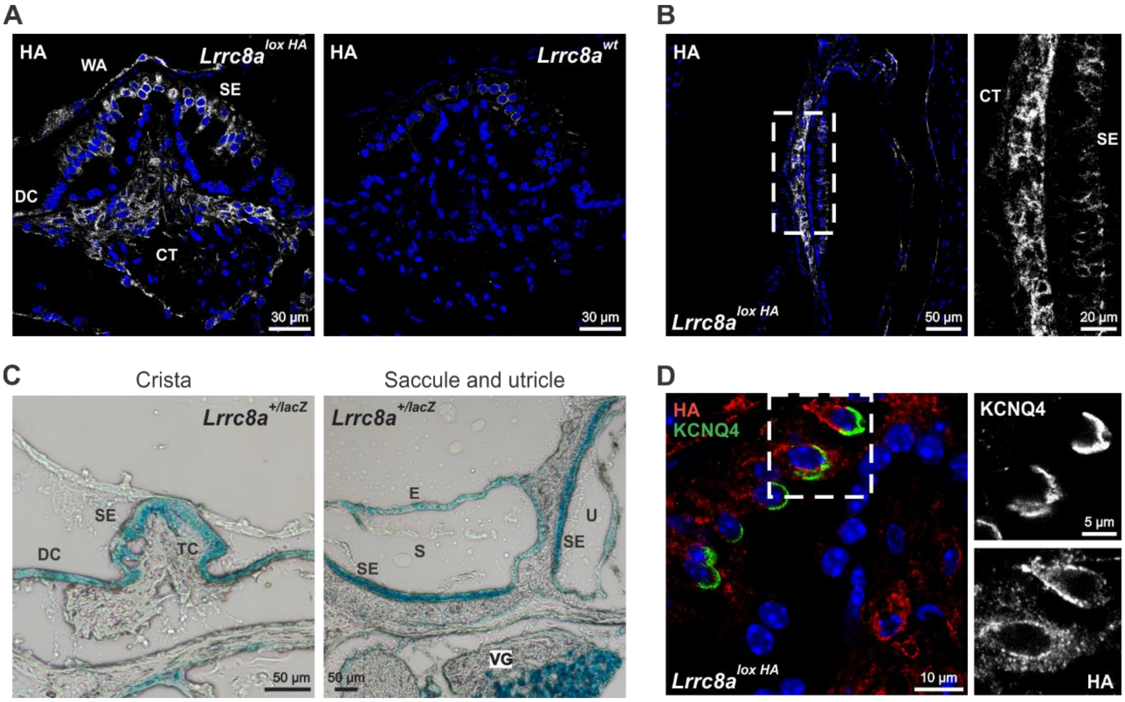

Figure S2. LRRC8A expression in vestibular organ. A-B. LRRC8A was detected by HA-epitope labeling in vestibular section of an 11-week-old *Lrrc8a*$^{lox\ HA/lox\ HA}$ mouse and compared to control WT mice. LRRC8A is present in the sensory epithelium (SE) and fibrocytes of connective tissue (CT) in the crista (**A**) and the utricle (**B**), and also in dark cells (DC) and the wall of ampulla (WA). **C.** LRRC8A expression as indicated by X-Gal staining of vestibular sections from 13-week-old *Lrrc8a*$^{+/lacZ}$ and WT control mice. Labeling is observed in the sensory epithelium (SE), transitional cells (TC) and dark cells (DC) of the crista (left panel), as well as in the sensory epithelia (SE) and the epithelium of the membrane labyrinth (E) in utricle (U) and saccule (S) (right panel). Labeling is also observed in the cells of the vestibular ganglion (VG). **D.** Higher magnification showing HA-staining of hair cells in the crista of a 11-week-old *Lrrc8a*$^{lox\ HA/lox\ HA}$ mouse. Punctate staining of the outer membrane and the cytoplasm. LRRC8A is almost absent from calyx synapses identified by KCNQ4 K^+-channels present in calyx terminals ensheathing type I hair cells (72). Images are collected from at least two different mice.

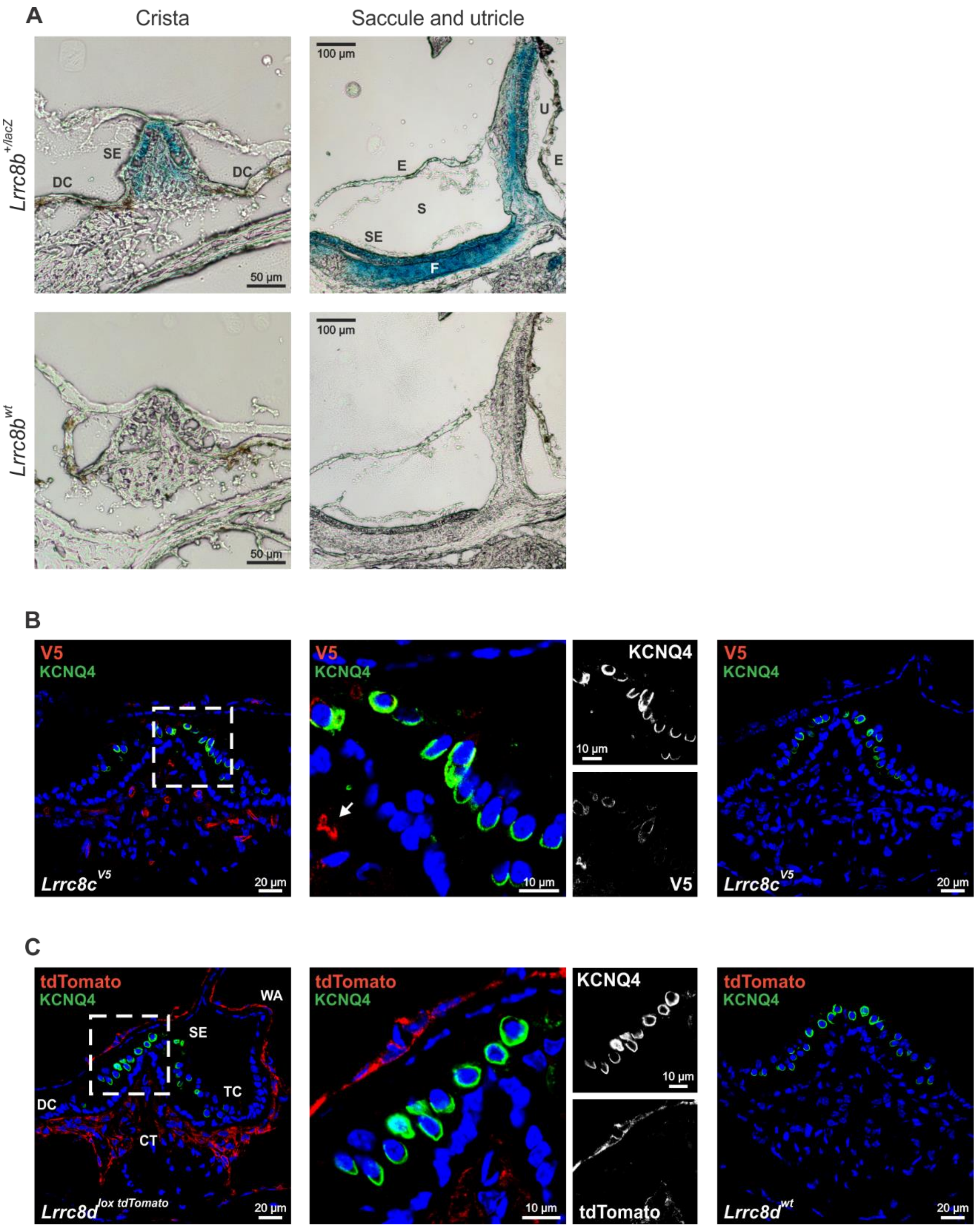

Figure S3. Expression of LRRC8B-E in the vestibular organ. A. X-Gal stained vestibular sections from 6-week-old *Lrrc8b*$^{+/lacZ}$ and control WT mice. β-Galactosidase is expressed in the sensory epithelia (SE) and fibrocytes (F) in the vestibular organ. No staining was detected in the epithelium of the membranous labyrinth (E) and dark cells (DC) in the crista. S, saccule; U, utricle. **B.** LRRC8C is weakly expressed in vestibular hair cells, whose calyx terminals are labelled with KCNQ4. Blood vessels strongly express

LRRC8C (marked by a white arrow). 12-week-old *Lrrc8c*$^{V5/V5}$ mice. **C.** LRRC8D could only be detected in fibrocytes in the connective tissue (CT) and the wall of ampulla (WA), but not in vestibular hair cells labelled at the calyx with KCNQ4. DC, dark cells; SE, sensory epithelium; TC, transitional cells. 9-week-old *Lrrc8c*$^{lox\ tdTomato/lox\ tdTomato}$ mice. Images are collected from at least two different ears.

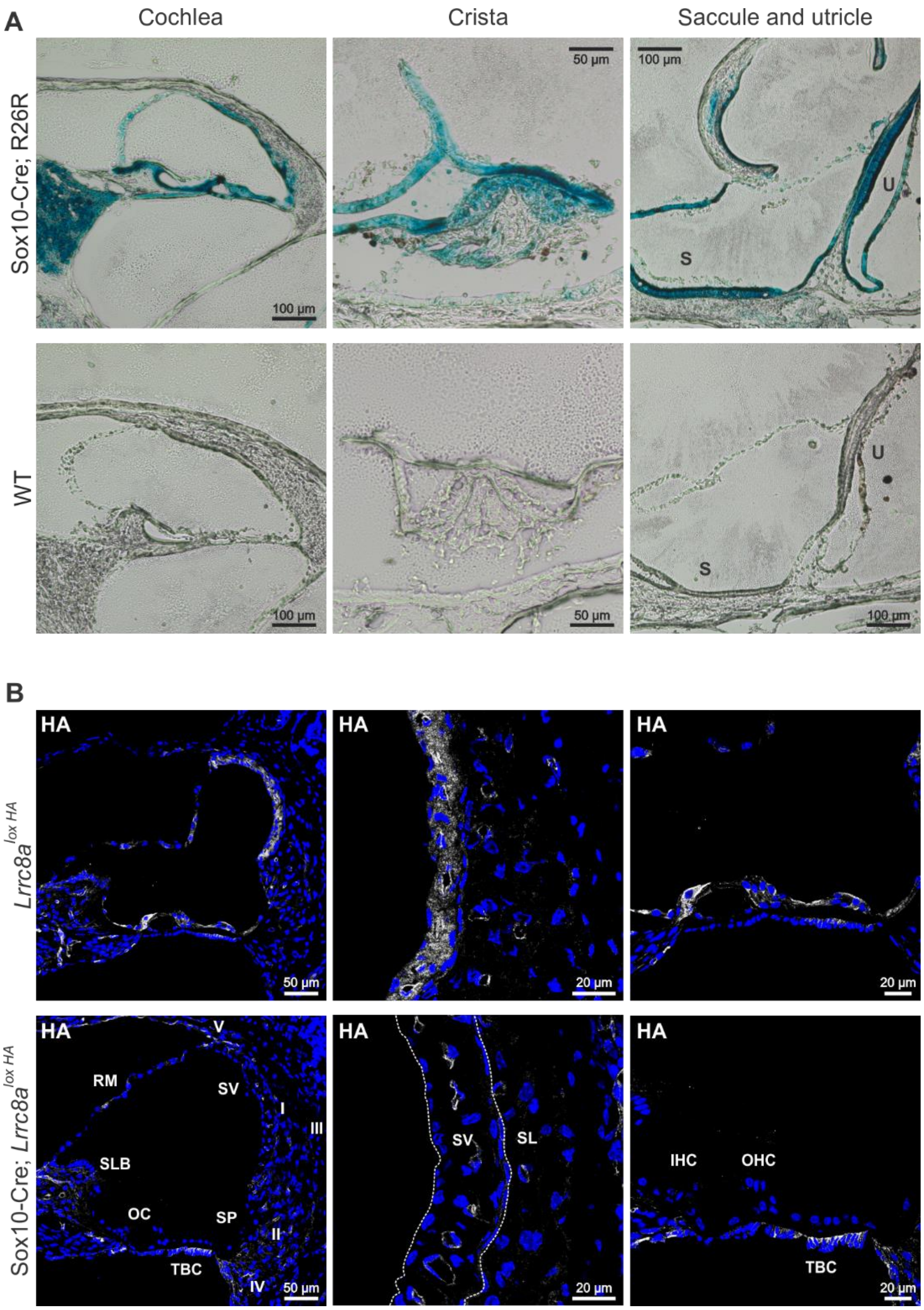

Figure S4. Specificity of cell-type specific *Lrrc8a* disruption under Sox10-Cre promotor. A. Expression pattern of Cre-recombinase inner ear in Sox10-Cre mice crossed to reporter mice. In in 20-week-old Sox10-Cre; R26R mice, X-Gal staining reveals Cre-recombinase expression in the organ of Corti, stria vascularis, spiral ganglion, Reissner`s membrane, and cells in outer sulcus and spiral prominence. In the crista, saccule (S) and utricle (U), the β-galactosidase is expressed in the sensory epithelia and vestibular membranes. **B.** Specificity of cell-type specific *Lrrc8a* disruption in Sox10-Cre; *Lrrc8a*$^{lox\ HA/lox\ HA}$ mice (P12). HA-tagged LRRC8A could not be detected in the stria vascularis (SV) and organ of Corti (OC). Vasculature

in the spiral limbus (SLB), stria vascularis (SV) and the spiral ligament (SL), few cells in Reissner's membrane (RM), type II and type IV fibrocytes and tympanic border cells (TBC) retained an HA signal. I-V fibrocytes type I to V; SP spiral prominence and outer sulcus region; IHC inner hair cells; OHC outer hair cells.

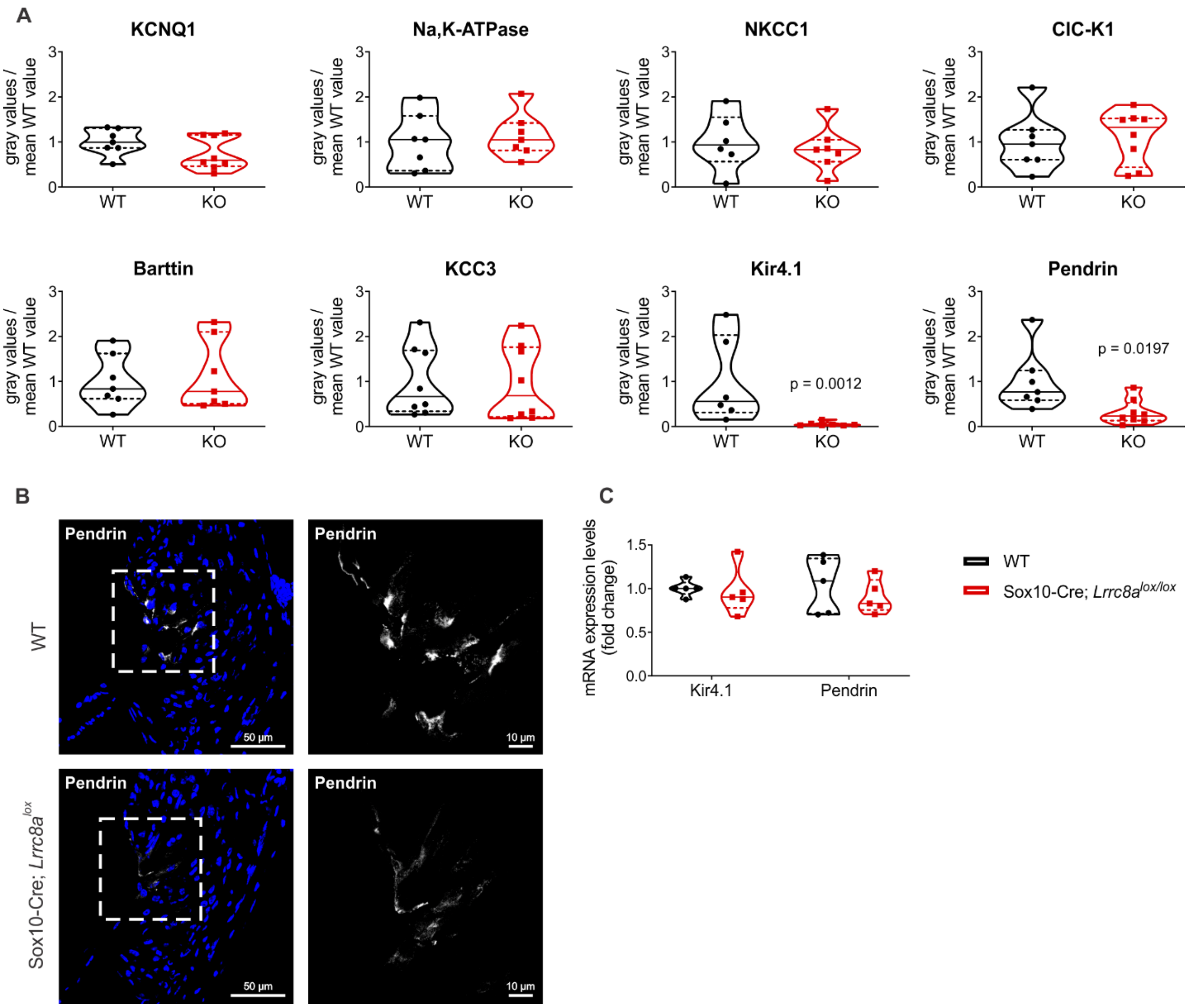

Figure S5. Changes in levels of ion transport proteins secondary to LRRC8A knockout. A. Quantification of transport proteins levels from immunohistochemistry. Kir4.1 and pendrin protein levels, but not those of other transporters of the stria vascularis (KCNQ1, ClC-K, NKCC1, barrtin, Na,K-ATPase) and spiral ligament (KCC3), are reduced in 16-20-days-old Sox10-Cre; *Lrrc8a*$^{lox/lox}$ compared to wild-type mice. Average gray values were divided by a mean value among wild-types, each point represent an average value across different inner ear slices of one mouse. Truncated violin plots, median is represented as a solid line, quartiles as broken line. Mann-Whitney test for Kir4.1, unpaired t test for pendrin. KCNQ1: WT N=7, KO N=8; Na,K-ATPase: WT and KO N=7, NKCC1: WT N=6, KO N=7; ClC-K1: WT N=7, KO N=8; Barrtin: WT and KO N=7; KCC3: WT and KO N=8; Kir4.1: WT N=6, KO N=7; Pendrin: WT N=7, KO N=8. **B.** Reduced expression of pendrin in the spiral ligament of 2-week-old Sox10-Cre; *Lrrc8a*$^{lox/lox}$ mice. **C.** Kir4.1 and pendrin mRNA levels are not changed in stria vascularis and spiral ligament of 1-year-old Sox10-Cre; *Lrrc8a*$^{lox/lox}$ mice. Data is shown as a fold change relative to the mean across all WT. Stria vascularis and spiral ligament were separated from the spiral ganglion, spiral limbus and organ of Corti; the purity of preparation was controlled through mRNA presence of strial marginal cell marker KCNQ1 and outer hair cell marker KCNQ4. Data is not significantly different (unpaired t test). N = 5 for both WT and KO.

Truncated violin plots, median is represented as a solid line, quartiles as broken line. N refers to the number of investigated mice.
